# Effects of interferons and double-stranded RNA on human prostate cancer cell apoptosis

**DOI:** 10.18632/oncotarget.5508

**Published:** 2015-10-01

**Authors:** Haiyan Tan, Chun Zeng, Junbo Xie, Norah J. Alghamdi, Ya Song, Hongbing Zhang, Aimin Zhou, Di Jin

**Affiliations:** ^1^ Institute of Cancer Stem Cell, Dalian Medical University, Dalian, China; ^2^ Clinical Chemistry Program, Department of Chemistry, Cleveland State University, Cleveland, OH, USA; ^3^ Center for Gene Regulation in Health and Diseases, Cleveland State University, Cleveland, OH, USA; ^4^ College of Biotechnology and Food Science, Tianjin University of Commerce, Tianjin, China; ^5^ College of basic medical sciences, Dalian Medical University, Dalian, China

**Keywords:** interferon, double-stranded RNA, apoptosis, prostate cancer, signaling

## Abstract

Prostate cancer is the second most commonly diagnosed cancer among men in the United States. Prostate cancer therapy is severely hampered by lack of response and development of resistance to conventional chemotherapeutic drugs in patients. Therefore, the development and discovery of new drugs have become an urgent clinical need. Interferons (IFNs), a family of pleiotropic cytokines, exert antitumor activities due to their anti-proliferative, immunomodulatory and proapoptotic functions. Here, we report that pretreatment of prostate cancer PC-3 cells with IFNs sensitized these cells to double-stranded RNAs (dsRNAs)-induced apoptosis. The enhancement effect of IFN treatment was dependent on IFN subtypes, in particular, IFN γ. In comparison with IFN α or β, IFN γ treatment remarkably augmented apoptosis in PC-3 cells induced with polyinosinic:polycytidylic acid (poly I:C), a synthesized form of dsRNA. We demonstrated that IFN-signaling was necessary for these effects by using mutant cell lines. Transfection of 2–5A, the activator of RNase L, or silencing of dsRNA-dependent protein kinase R (PKR) by siRNA did not have any significant impact on this event, suggesting that neither RNase L nor PKR was involved in poly I:C/IFN γ-induced apoptosis in the cells. Further investigation of the apoptotic pathway revealed that Bak, a pro-apoptotic member of the Bcl-2family, was synergistically up-regulated by IFN γ and poly I:C, whereas other members of the family were not affected. Knocking down of Bak demonstrated its contribution to poly I:C/IFN γ-induced apoptosis in the cells. We believeour findings will precipitate the design of novel therapeutic strategies for prostate cancer.

## INTRODUCTION

In developed countries, prostate cancer is the second most frequently diagnosed cancer, which affects about one in six men in the United States. Although preventative steps such as prostate specific antigen (PSA) screening and early detection have been undertaken to reduce the mortality, it is nevertheless still the second leading cause of cancer death in men [1].

Treatment for prostate cancer depends on the cancerous stage of the patient. For older patients with slow growing tumors, watchful waiting may be suggested. In the case of young patients and fast growing tumors, the most common treatment is radical prostatectomy and transurethral resection of the prostate. Radiation therapy, a treatment with high-energy rays which kills cancer cells, is applied as the first treatment for low-grade cancer that has not spread outside the prostate gland or has only spread to nearby tissue. Cryosurgery that freezes the prostate cancer cells with cold metal probes can also be used for prostate cancer without metastasis. However, for men with metastatic disease, androgen-ablation therapy termed as hormone therapy is the standard initial treatment. This treatment decreases the level of androgen, which compels prostate cancer shrink or delay growth. Unfortunately, most patients eventually become resistant to hormone therapy. Hormone-refractory prostate cancer (HRPC) is a common condition that leads to significant morbidity and mortality [2]. Cytotoxic chemotherapy has been considered as the best way to treat HRPC. Docetaxel, a member of taxane compounds classified as anti-microtubular agents, has become the first-line chemotherapeutic drug for this disease [3, 4]. However, lack of response and development of resistance to docetaxel in a considerable number of patients limits its applications in prostate cancer therapy [5, 6]. Therefore, the development and discovery of new drugs capable of prolonging survival of patients with prostate cancer have become an urgent clinical need.

IFNs are a family of cytokines expressed in eukaryotic cells as an early response to stimuli, such as viral infections, dsRNA and immune inducers [7]. There are two major classes of IFNs: Type I and type II. Type I IFNs consist of several subtypes, mainly IFN α and IFN β, and are induced in most cell types by viruses and dsRNA. In contrast, type II IFN is induced potently in T lymphocytes and natural killer (NK) cells in response to immune and inflammatory stimulation [8]. Most recently, a new type of IFN-like protein has been identified and classified as Type III IFN or IFN λ. Despite the differences in their amino acid sequences, all three types of IFNs display similar biological responses through the induction of IFN-stimulated gene (ISGs) expression [9, 10].

IFN α is the first IFN used in the treatment of cancer. Treatment with IFN α results in significant clinical outcomes and prolongs survival of patients with malignant hematological diseases, such as hairy cell leukemia, myeloma and lymphoma [11, 12]. Furthermore, clinical trials have demonstrated that IFN α also exerts its therapeutic mechanisms against other tumor types, including renal cell carcinoma, Kaposi's sarcoma, and melanoma [13]. Pre-clinical studies have shown that IFNs are able to inhibit the growth of prostate cancer cells. For example IFN β increases the expression of androgen receptors, improving adhesion potential of androgen-insensitive prostate cancer cells [14], while adenovirus-mediated delivery of IFN γ gene can inhibit the growth of prostate cancer cells *in vitro* and xenografts *in vivo* [15]. However, the role of IFNs in the treatment of prostate cancer is understudied, particularly in its clinical applications. The limited application is probably due to the lack of efficacy and cytotoxicity in prostate cancer patients [16–18].

The antitumor activity of IFNs is believed to be, at least in part, through inducing apoptosis in cancer cells. Type I and Type II IFNs are able to effectively induce apoptosis in a wide range of malignant cell types, such as herpes-associated lymphomas, acute promyelocytic leukemia (APL), non-small-cell lung cancer, non-melanoma skin cancer and glioma [19]. IFNs have been reported to induce cell apoptosis through the activation of the death receptor cascade. The induction of TRAIL and/or Fas/FasL in response to IFNs leads to recruitment and activation of FADD. FADD activation, in turn, activates caspase-8, initiating activation of the caspase cascade. On the other hand, IFNs also induce caspase 4 and caspase-8. Activated caspase-8 cleaves Bid, a proapoptotic member of Bcl-2 family, resulting in disruption of mitochondrial potential and the release of cytochrome C from the mitochondria into the cytoplasm. Here, it acts as a cofactor to stimulate the complexion of Apaf1 with caspase-9, subsequently activating caspase-3. A variety of ISGs including the members of the IFN regulatory factor (IRF) family, dsRNA dependent protein kinase (PKR), 2–5A dependent RNase L (RNase L), TNF-related apoptosis-inducing ligand (TRAIL), promyelocytic leukemia gene (PML) and the death associated proteins (DAPs) exert their tumor suppressing functions through the induction of apoptosis in tumor cells [19]. Interestingly, the involvement of different ISGs in IFN-induced apoptosis depends on cell types. For example, TRAIL and XIAP associated factor 1(XAF1) are believed to contribute to IFN-induced apoptosis in melanoma cells, whereas an induction of the regulators of IFN-induced death (RIDs) is necessary in IFN-induced ovarian carcinoma cell apoptosis [20–22]. Selective inhibition of one or more apoptotic ISGs, or the acquisition of defects in IFN-signal transduction components increases the survival of cancer cells.

In this study, we found that IFNs, especially IFN γ, enhanced the vulnerability of prostate cancer cells to poly I:C-induced apoptosis. Further mechanistic studies demonstrated that the IFN signaling pathway was necessary for this event and poly I:C/IFN γ inducing prostate cell apoptosis was partially through upregulating the Bak expression. Our findings may provide insight for a possible application in prostate cancer therapy.

## RESULTS

### IFN γ and dsRNA synergistically decrease the viability of PC-3 cells

**Figure 1:**
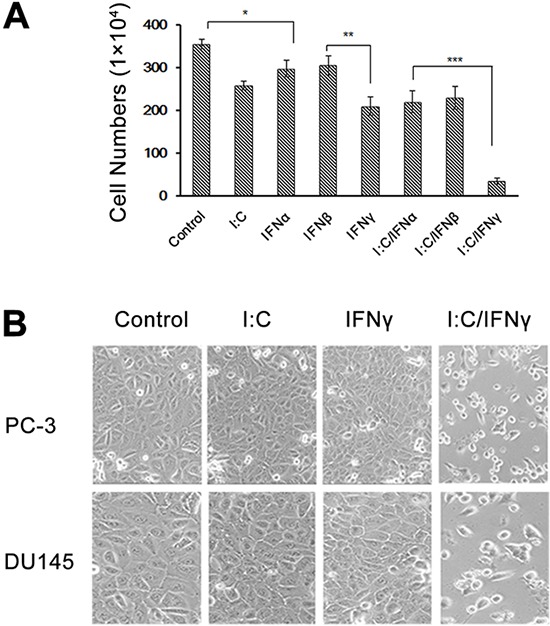
Effect of poly I:C and IFNs on PC-3 cell viability PC-3 cells were treated with 1,000 unit/ml IFN α, β or γ overnight and transfected with and without 1 μg/ml poly I:C in the presence of lipofectamine. **A.** The viable cells were analyzed by trypan blue exclusion assays and the cell numbers were averaged from three independent experiments. Error bars represent ±SEM, and Student's *t* test was used. *p < 0.05, **p < 0.01, ***p < 0.001; and **B.** The pictures were taken under Olympus CKX31 at 100 × magnification.

The antiproliferative effect of IFNs has been well established [7]. To determine the direct effect of IFNs on prostate cancer cells, we treated PC-3 cells, a prostate adenocarcinoma cell line, with and without IFN α, β or γ and then determined the growth of the cells. Interestingly, we found that IFN γ displayed an overt inhibitory effect on PC-3 cells when compared with IFN α and β. In recent years, studies have revealed that a combination of IFNs with cytotoxic compounds, such as paclitaxel and thalidomide, augments the cytotoxicity for prostate cancer cells and renal cell cancer in an additive manner [23, 24]. DsRNA is a side-product of viral infection, which is an effective activator for several IFN-inducible enzymes, and mediates the IFN action in antiviral infection and anti-cellular proliferation. To determine the effect of dsRNA on prostate cancer cells, we pre-treated PC-3 cells with and without IFNs for 12 hours and incubated the cells with poly I:C, a type of synthesized dsRNA, for 48 hours. Surprisingly, we found that only IFN γ and poly I:C synergistically induced a remarkable inhibitory effect on PC-3 cells as shown in Fig. 1A. IFN α, β, γ or poly I:C alone only slightly suppressed the growth of PC-3 cells. However, pre-treatment of these cells with individual IFN, followed by poly I:C, significantly reduced the cell viability. Specifically only about 10% cells survived after pre-treated with IFN γ and incubated with poly I:C for 48 hours. The cell morphological images could be visible under an Olympus CKX31 microscope at 100 X magnification (Fig. 1B). To rule out any bias caused by cell lines, we similarly treated DU145 cells, another prostate cancer cell line, and obtained comparable results (Fig. 1B and S1A). These results suggest that prostate cancer cells primed with IFN γ are more vulnerable to poly I:C-induced death. To determine the effect of a combination of IFNs with poly I:C on other cell types, we used the same method to treat H522 (human non-small cell lung cancer), RCC45 (human renal cell carcinoma) and SK-HEP-1 (human liver adenocarcinoma) cells. Interestingly, the synergistic role of IFN was cell type dependent. As shown in Fig. S1B–S1D, H522 and Sk-Hep-1 cells were significantly sensitized to poly I:C after the cells were primed with IFN α/β, not IFN γ,while RCC45 cells could be primed by all three types of IFNs.

### Poly I:C/IFN γ induces apoptosis in prostate cancer cells

**Figure 2:**
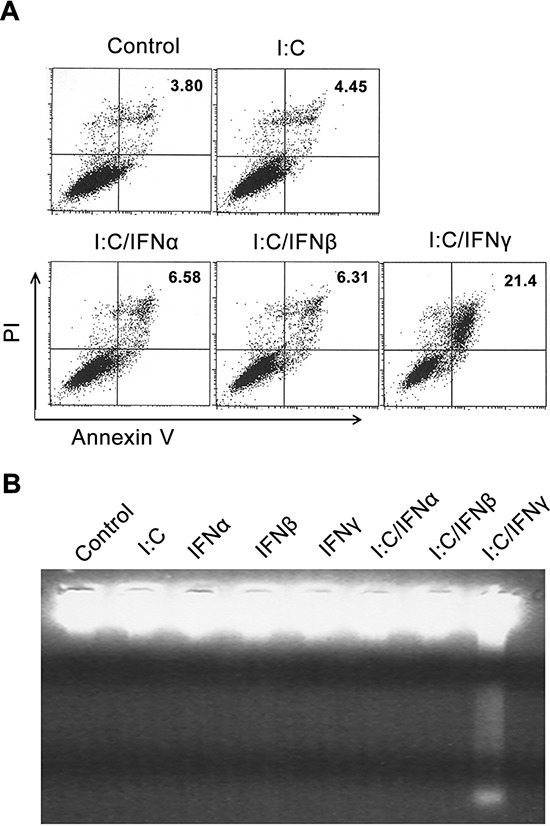
Annexin-V FACS analysis and DNA Fragmentation PC-3 cells were treated with either poly I:C or poly I:C in combination with subtypes of IFNs in the presence of lipofectamine for 24 hours. **A.** Apoptotic cells were analyzed by Annexin V assays. **B.** The DNA fragments were purified using an Apoptotic DNA Ladder Kit, separated by 2% agarose gel electrophoresis, and stained with ethidium bromide.

To determine if the inhibition of prostate cancer cell growth resulted from apoptosis, an Annexin V assay was performed to evaluate poly I:C/IFN γ-induced PC-3 cell death. In this experiment, PC-3 cells were treated with and without 1 μg/ml of poly I:C alone, or combined with 1,000 units/ml of IFN α, β or γ. After 24 hours of incubation, the cells were subjected to an Annexin V assay. As shown in Fig. 2A, 21% of PC-3 cells underwent apoptosis after poly I:C/IFN γ treatment, whereas poly I:C alone, poly I:C/IFN α and poly I:C/IFN β only resulted in 4.45%, 6.58% and 6.31% apoptotic cells, respectively, suggesting that poly I:C/IFN γ is a potent inducer of PC-3 cell apoptosis. We also performed experiments to determine the dose response and found that poly I:C at a concentration of 100 ng/ml and IFN γ at 50 units/ml were able to induce a full response in these cells, suggesting their potential in clinical application. However, WI-38 cells, a normal human lung fibroblast cell line, and PrECs cells, a normal prostate epithelial cell line, did not respond to the same treatment. A significantly higher concentration of poly I:C (>10 times) was needed to induce apoptosis in both types of normal cells, indicating that poly I:C/IFN γ selectively induces prostate cancer cell apoptosis (data not shown). To further evaluate poly I:C/IFN γ-induced apoptosis in PC-3 cells, DNA fragmentation in the cells after treatment was examined. As shown in Fig. 2B, DNA fragmentation was only observed in the cells treated with poly I:C/IFN γ, confirming poly I:C/IFN γ-induced apoptosis.

**Figure 3:**
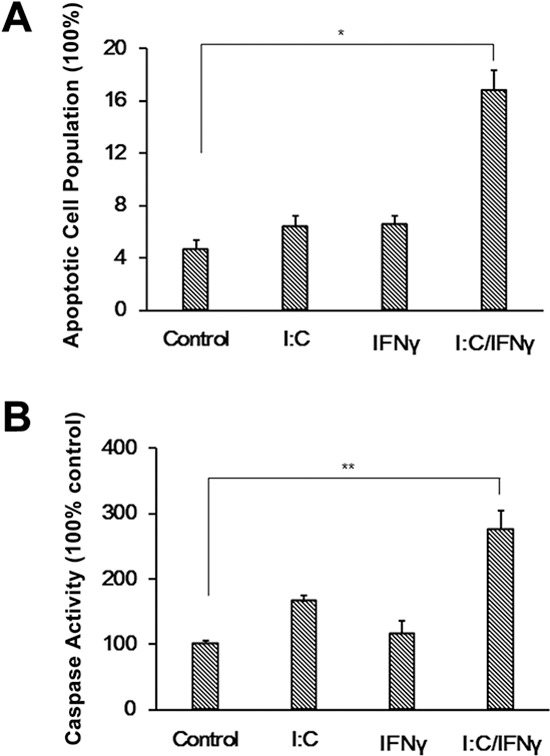
TUNEL assay and induction of the caspase 3 activity **A.** PC-3 cells were treated with poly I:C, IFN γ alone, or a combination of both in the presence of lipofectamine. After incubation for 24 hours, the cells were subjected to a TUNEL assay by using an apo-Brdu kit. The population of apoptotic cell numbers was averaged from two independent experiments. Error bars represent ± SEM, and Student's *t* test was conducted. *p < 0.05. **B.** PC-3 cells were treated as described above. The activity of caspase 3 was measured using a Caspase-GloTM 3/7 assay kit. Experiments were performed two times in triplicates. Error bars represent ± SEM, and Student's *t* test was used. **P < 0.01.

To further demonstrate poly I:C/IFN γ induced apoptosis, the cells after treatment were subjected to a terminal deoxynucleotidyl transferase dUTP nick end labeling (TUNEL) assay. As expected, the population of apoptotic cells was nearly 4-fold higher in the cells treated with poly I:C/IFN γ compared to the control (Fig. 3A). We also evaluated the activity of caspase 3 in PC-3 cells after treatment with poly I:C/IFN γ. The activity of caspase 3 in the cells treated with poly I:C/IFN γ was examined by using a Caspase-GloTM 3/7 assay kit (Promega). IFN γ and poly I:C alone slightly induced the activity of caspase 3 in PC-3 cells. However, the synergistic role of IFN γ and poly I:C was evident. Poly I:C/IFN γ induced an increase of caspase 3 activity by about 3 folds (Fig. 3B). This observation was also confirmed in DU145 cells (Fig. S2).

### IFN signaling is necessary for poly I:C/IFN γ-induced apoptosis in prostate cancer cells

**Figure 4:**
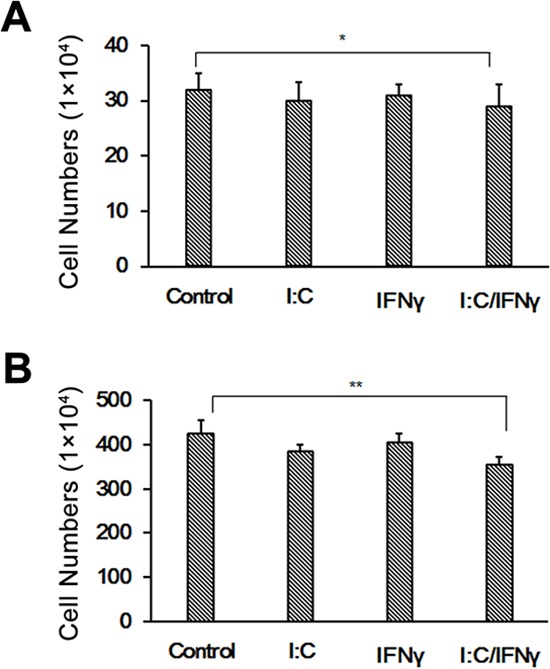
Effect of poly I:C and IFN γ on LNCaP and U3A cell viability LNCaP **A.** and U3A **B.** cells were treated with 1 μg/ml of poly I:C, 1,000 units of IFN γ alone, or a combination of both in the presence of lipofectamine. After incubation for 48 hours, the viable cells were analyzed by trypan blue exclusion assays and the cell numbers were averaged from two independent experiments. Error bars represent ± SEM, and Student's *t* test was conducted. *p < 0.05; **p < 0.01.

The gene expression mediated by IFNs is through the activation of the JAK/STAT pathway. To determine if IFN signaling is necessary for poly I:C/IFN γ-induced apoptosis in prostate cancer cells, we used LNCaP cells to perform the experiment. The LNCaP cell line is an altered prostate cancer cell line deficient of JAK1, a key component in the JAK/STAT pathway [25]. As we expected, LNCaP cells were unresponsive to poly I:C/IFN γ in the same condition for PC-3 cells as shown in Fig. 4A. However, the cells slightly underwent apoptosis at a concentration of 25 μg/ml poly I:C (data not shown), which may be caused by activation of other pathways [26]. To further strengthen our finding, we performed the same experiment by using U3A cells in which STAT1 is mutated. A similar result was obtained (Fig. 4B). Taken together, these results suggest that the JAK/STAT pathway of IFNs is necessary for the synergistic effect of poly I:C/IFN γ on prostate cancer cell apoptosis.

### PKR and RNase L is not involved in the event

**Figure 5:**
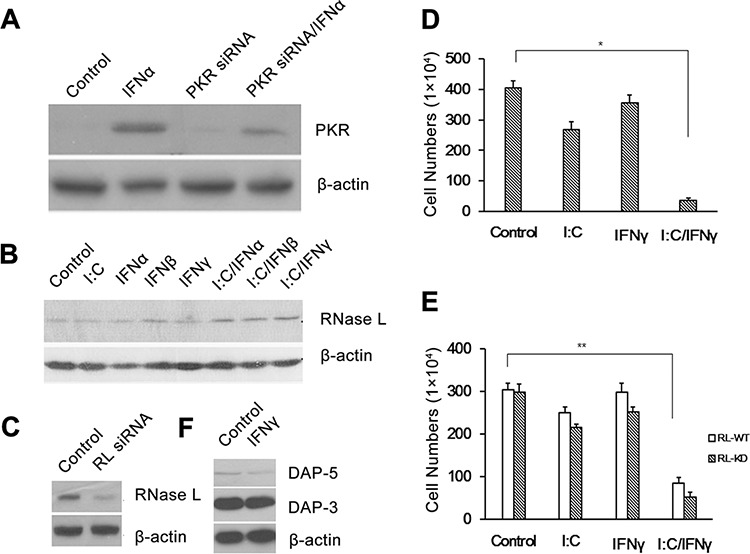
Effect of PKR and RNase L on PC-3 cell apoptosis PKR **A.** and RNase L **C.** were knocked down in PC-3 cells. PKR **D.** and RNase L **E.** knock down cells were then treated with poly I:C, IFN γ alone, or a combination of both. The viable cells were analyzed by trypan blue exclusion assays and the cell numbers were averaged from three independent experiments. Error bars represent ± SEM, and Student's *t* test was used. *p < 0.05; **p < 0.01. **B.** The expression of RNase L was induced by different treatments. **F.** The expression of the DAP members in PC-3 cells was induced by IFN γ.

DsRNA-dependent protein kinase (PKR) is a serine/threonine kinase induced in mouse and human cells by IFNs and requires dsRNA for its activity. PKR displays a broad range of biological activities including inhibition of cell growth and induction of apoptosis [9]. To determine whether PKR is involved in the apoptosis of PC-3 cells induced by poly I:C/IFN γ, PKR in the cells were knocked down by PKR siRNA (Fig. 5A) and apoptosis induced by poly I:C/IFN γ was determined. PKR was not found to contribute to poly I:C/IFN γ-induced apoptosis in PC-3 cells (Fig. 5D).

RNase L is a key enzyme in the 2–5A system of IFN against virus and cell proliferation. 2′–5′ linked oligoadenylates known as 2–5A, the activator of RNase L, is synthesized by 2–5A synthetases requiring dsRNA for their activities. The role of RNase L in cell apoptosis has been well established [27]. To determine whether RNase L is involved in poly I:C/IFN γ-induced prostate cancer cell apoptosis, we first analyzed the expression of RNase L in the cells treated with IFNs and individual IFN with poly I:C. As shown in Fig. 4B, the level of RNase L in the cells was not significantly changed, no matter if the cells were treated with individual IFN alone or combined with poly I:C. RNase L in the cells was knocked down by using siRNA (Fig. 5C) and the cells were then treated with poly I:C or IFN γ alone, or a combination of both. The cell viability was not affected in the presence or absence of RNase L (Fig. 5E). These results suggest that the 2–5A system is not involved in this event.

It has been reported that death associated proteins (DAPs) such as DAP-3 and 5 contributes to IFN γ-induced apoptosis in different cell types [28]. To determine if DAPs are the effectors in poly I:C/IFN γ-induced apoptosis in prostate cancer cells, we examined the expression of DAP3 and 5 by Western blot analysis in the cells after IFN γ treatment. The expression of DAP 3 and 5 was clearly IFN γ independent (Fig. 5F) in PC-3 cells, suggesting the DAP proteins may not be active in poly I:C/IFN γ-induced apoptosis.

### Bak partially contributes to poly I:C/IFN γ-induced apoptosis

**Figure 6:**
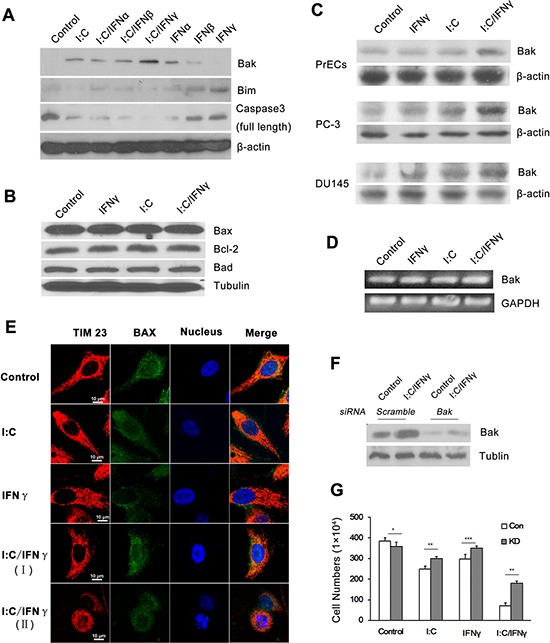
Expression of apoptosis-associated genes in prostate cancer cells after treatment **A.** PC-3 cells were treated with 1,000 units/ml of individual IFN alone, or in combination with 1 μg/ml of poly I:C in the presence of lipofectamine. The expression of several apoptosis associated proteins was determined by Western blot analysis using antibodies to Bak, Bim and the full length of caspase 3. **B.** PC-3 cells were treated with 1 μg/ml of poly I:C, 1,000 units/ml of IFN γ alone, or a combination of both in the presence of lipofectamine for 14 hours. The expression of Bax, Bcl-2 and Bad was examined by Western blot analysis. **C.** PC-3, DU-145 and PrECs cells were treated as described above and the expression of Bak in the cells was examined by Western blot analysis. **D.** PC-3 cells were treated as described above. Total RNAs were isolated by using the Trizol Reagent (Invitrogen, Grand Island, NY) and the expression of Bak was determined by RT-PCR. The PCR primers for Bak are: sense 5′-CTG CCC TCT GCT TCT GAG GA-3′ and antisense 5′-CTGTCA GGA TGG GAC CAT TG-3′ **E.** Immunofluorescent staining of Bax in PC-3 cells after treatment. I:C/IFN γ **I.** pre-apoptotic cell; I:C/IFN γ (II): apoptotic cell. Tim 23: red (PE); Bax: green (FITC); and nucleus: blue (Hoechst). **F.** The expression of Bak in PC-3 cells was knocked down by through the utilization of a siRNA kit. **G.** Control and knock down cells were treated with 1 μg/ml of poly I:C, 1,000 units of IFN γ alone, or a combination of both in the presence of lipofectamine for 48 hours. The viable cells were analyzed by trypan blue exclusion assays and the cell numbers were averaged from three independent experiments. Error bars represent ± SEM, and Student's *t* test was used. *p < 0.05; **p < 0.01 and ***P < 0.001.

There are a variety of gene products involved in the process of cell apoptosis. To explore if poly I:C/IFN γ treatment impacts the expression of certain pro- or anti-apoptotic proteins in the cells, we examined the level of some members in the Bcl-2 family. As shown in Fig. 6A and 6B, a combination of IFN γ and poly I:C had no significant impact on the expression of Bax, Bad, Bim and Bcl-2 in PC-3 cells. However, the treatment synergistically enhanced the expression of Bak, a pro-apoptotic member of the Bcl-2 family [29]. The significantly increased Bak expression in DU145 was also observed upon the treatment, although poly I:C/IFN γ was slightly enhanced the level of Bak in PrECs cells, a normal prostate cell line (Fig. 6C). Overtly, poly I:C/IFN γ induced the expression of Bak at its transcriptional level (Fig. 6D). Bax is mainly found in the cytosol. Upon apoptotic stimulation, Bax undergoes a conformational shift and becomes organelle membrane-associated, in particular, mitochondrial membrane associated. To further demonstrate if Bax is involved in the event, despite its unchanged level, we performed immune staining to localize Bax in the cells under different conditions. As shown in Fig. 6E, the location of Bax in the cell was not obviously altered in the presence or absence of these stimuli, confirming that Bax did not contribute to poly I:C/IFN γ-induced apoptosis in PC-3 cells.

Bak is a pro-apoptotic member in the Bcl-2 family, which is also involved in a wide variety of cellular activities [30]. To determine if the increased expression of Bak plays a major role in poly I:C/IFN γ-induced apoptosis, we first knocked it down by using siRNA in PC-3 cells. The cells were then treated with poly I:C/IFN γ. As shown in Fig. 6F and 6G, knocking down of Bak significantly reduced poly I:C/IFN γ-induced apoptosis, suggesting that Bak may be the key effector in this event.

## DISCUSSION

There is an urgent need for new drugs to treat patients with hormone-refractory prostate cancer (HRPC). Although clinical trials by using either IFN γ or poly I:C alone have been initiated for treating several types of cancer, the toxicity at high doses severely hindered the outcomes [11, 12]. In this study, we report that a combination of IFN γ with poly I:C was able to induce apoptosis in prostate cancer cells at a significant lower dose and Bak, a pro-apoptotic member in the Bcl-2 family, may be the effector in the event. Our results may provide a novel therapeutic approach to treat patients with HRPC.

Cancer therapy using a single drug is often not sufficient to elicit a significant therapeutic response. Thus, a combination of different therapeutic agents is becoming one of the most common strategies used in oncology. Preclinical data and clinical trials have demonstrated that such a combination can enhance anti-tumor activity of these compounds and decrease their toxicities. For example, IFN α is combined with chemotherapeutic drugs such as sorafenib, temsirolimus and bevacizumab to treat renal cell carcinoma [31, 32]. Additionally, a phase II trial indicates that a combination of 5-fluorouracil (5-FU), cisplatin and IFN α results in a high response rate in advanced esophageal squamous cell carcinoma (SCC) [33]. Furthermore, a phase I/II clinical trial shows that IFN γ can be used in combination with carboplatin and paclitaxel as a safe and effective first-line treatment option for ovarian cancer [34]. We expect that our strategy will provide a novel treatment for prostate cancer.

RNas L and PKR are IFN-inducible proteins mediating IFN functions against viral infection and cell proliferation at the transcriptional and translational levels. The activation of RNase L and PKR is dsRNA-dependent, although the role of dsRNA in the RNase L function is indirect. A line of evidence has shown that RNase L and PKR play an important role in cell apoptosis as well [9]. PKR is clearly not involved in poly I:C/IFN γ-induced apoptosis in prostate cancer cells since there was no change in apoptotic cells after knocking down of PKR in the cells. 2–5A is the activator of RNase L, which can be produced by 2–5A synthetases, a family of IFN-induced and dsRNA dependent enzymes. Transfection of 2–5A into cells results in apoptosis in several cell types through activation of RNase L [27]. Apoptosis is induced in PC-3 cells by 2–5A, but not in the cells in which RNase L was knocked down [35]. However, poly I:C/IFN γ was able to induce apoptosis at a similar extent in PC-3 cells with or without RNase L, suggesting that RNase L does not contribute to the event.

The Jak-Stat pathway mediates the function of IFNs [9]. Our results showed that the Jak-Stat pathway is necessary for poly I:C/IFN γ-induced apoptosis, since U3A cells in which STAT1 is mutated did not respond to poly I:C/IFN γ. Furthermore, poly I:C/IFN γ at the concentration we used was unable to induce apoptosis in LNCaP cells, an altered prostate cancer cell line deficient of JAK1. Interestingly, although all three types of IFN can synergistically enhance apoptosis induced by poly I:C, the effect is dependent on cell type. For example, H522 and Sk-Hep-1 cells were significantly sensitized to poly I:C after the cells were primed with IFN α/β, but not IFN γ, despite the fact that IFN α functioned better in H522 cells and IFN β was slightly more effective in Sk-Hep-1 cells, and RCC45 cells could be primed by all three types of IFNs (Fig. S1B–S1D). Hey1B cells, a human ovarian cancer cell line, were similar to PC-3 cells under the same condition (data not shown). These observations suggest that either IFNs may induce certain specific proteins, which play a distinctive role in dsRNA-induced apoptosis across different cell types. Further investigation of these mechanisms is warranted.

The Bcl-2 family plays an important role in apoptosis. A line of evidence has shown that apoptosis induced by IFN alone or combined with other agents is associated with altered levels of Bcl-2 family members. For example, IFN α2a induces apoptosis in OVCAR3 cells through Bak activation and translocation of AIF from the mitochondria to the nucleus [29]. In addition, IFN α with sorafenib together downregulates the expression of Mcl-1, Bcl-2 and Bcl-xL, resulting in hepatocellular carcinoma (HCC) cell apoptosis [36]. IFN γ also enhances apoptosis in macrophages under trophic stress by upregulation of p53 and Bax, and down regulation of Bcl-xl [37], while IFN γ combined with TNF-α induces pancreatic β-cell apoptosis via STAT1-mediated Bim activation [38]. Recently Riccioli et al. reported that poly I:C alone with lipofectamine was able to induce apoptosis in prostate cancer cells through activation of the TLR3/IRF3 pathway or PKC-α [26, 39, 40]. In our study, Bak may be one of the major contributors in poly I:C/IFN γ-induced apoptosis.

## MATERIALS AND METHODS

### Reagents and antibodies

Antibodies were purchased from different companies: Bim, Bax, Tim23, PKR and the full length of caspase 3 from BD Bioscience (San Jose, CA); β-actin, Tubulin, Bcl-2, Bad and Bak were from Santa Cruz Biotechnology, Inc. (Santa Cruz, CA). DAP3 and 5 antibodies were purchased from Cell Signaling (Danvers, MA). Human RNase L monoclonal antibody was a gift from Dr. Robert Silverman (Cleveland Clinic, OH).

### Cell culture and treatment

PC-3 human prostate cancer cells (ATCC, Manassas, VA) were grown in RPMI-1640 (Core Facility, Cleveland Clinic, Cleveland, OH) and supplemented with 10% fetal bovine serum (Biosource, Camarillo, CA) as well as antibiotics in a humidified atmosphere of 5% CO_2_ at 37°C. The cells were grown to 90% confluence and incubated with 1,000 units/mL of IFN α, IFN β and IFN γ (R & D Systems, Minneapolis, MN) overnight, and transfected with or without 1 μg/mL polyinosinic: polycytidylic acid (poly I:C) (Sigma, St. Louis, MO). The cell viability was analyzed by trypan blue exclusion assays.

### Annexin V assay

The Annexin V assay was performed using an Annexin V-FITC/propidium iodine apoptosis detection kit (BD Biosciences, San Jose, CA). Briefly, the cells treated with poly I:C alone or in combination with individual IFN for 24 hours were scraped and centrifuged at 1,000 × g for 10 min at 4°C, washed with ice cold PBS, and then re-suspended in 1 × binding buffer, provided by the manufacturer at a concentration of 1 × 10^6^/ml. FITC-Annexin V (5 μl) and propidium iodide (5 μl) were added to 100 μl of the cell suspension and the cells were incubated at room temperature for 15 min in the dark. After incubation, 400 μl of 1 × binding buffer was added to the cell suspension and the cells were analyzed by two color cytometry using a FACScan^™^ (Becton Dickinson, Franklin Lake, NJ).

### Determination of DNA fragmentation

DNA in the cells after treatment was isolated using an Apoptotic DNA Ladder Kit (Roche, Indianapolis, IN). Briefly, cell suspension (200 μl) in PBS was mixed with 200 μl of binding buffer. After incubation for 10 min at room temperature, 100 μl of isopropanol was added to the sample and mixed by vortexing. DNA was then purified through the use of glass fibers. DNA samples were separated on a 2% agarose gel and visualized with ethidine bromide staining under UV light.

### Caspase assays

The activity of caspase 3 in the cells treated with poly I:C or IFN γ alone, or a combination of both, was examined by using the Caspase-GloTM 3/7 reagent (Promega, Madison, WI). In brief, cytosolic extracts were prepared by suspending cell pellets in NP-40 lysis buffer (10 mM Tris-HCl, pH8.0, 5 mM Mg(OAc)_2_, 90 mM KCl, 0.2 mM PMSF, 100 units/ml aprotinin, 10 μg/ml leupeptin and 2% NP-40). After centrifugation at 10,000 g for 10 min, the cell extracts containing 40 μg proteins were transferred into a 96-well plate to mix with 50 μl of the Caspase-Glo3/7 reagent. After incubation for 1 hour at 37°C, caspase activity was then determined by a fluorescent plate reader (Dynex Technologies, Chantilly, VA).

### TUNEL assay

Terminal deoxynucleotidyltransferase (TdT) dUTP nick end labeling analysis (TUNEL) for DNA degradation was performed using an apo-Brdu kit (BD Biosciences, San Jose, CA). Cells after treatment were scraped using a scraper with the media containing floating cells. Cells were fixed in 1% paraformaldehyde for 30 min and stored in 70% ethanol at −20°C until staining and analysis. DNA fragmentation was examined by incorporating 5-bromo-2′-deoxyuridine and stained with a labeled anti-bromodeoxyuridine monoclonal antibody. The total DNA content was determined with propidium iodine, and the labeled cells were sorted by using a FACScan^™^ (Becton Dickinson, Franklin Lake, NJ).

### Western blot analysis

After treatment, cells were washed twice with ice-cold PBS and collected with a scraper. The cytoplasmic extracts were prepared as described above. Proteins (100 μg per sample) were fractionated on a SDS-10% polyacrylamide gel and transferred to the PVDF membrane (Millipore, Billerica, MA). The membrane was blocked with 5% nonfat milk in PBS containing 0.02% sodium azide and 0.2% (v/v) Tween 20, and incubated with different primary antibodies for 1 hour at room temperature. The membrane was then washed with PBS containing 0.2% (v/v) Tween 20 and incubated with specific secondary antibodies conjugated with horseradish peroxidase (Cell Signaling, Billerica, MA) for 1 hour at room temperature. After washing, proteins were detected by a chemiluminescent method according to the manufacturer's specification (Pierce, Rockford, IL).

### Immunofluorescence staining

PC3 cells were plated onto 12-mm diameter round glass cover slips in a six-well plate. After treatment, cells were washed three times with PBS and fixed with 3.7% paraformaldehyde for 10 min at 37°C. Fixed cells were rinsed in PBS, sequentially permeabilized with 0.2% Triton X-100 on ice for 10 min, and washed with PBS at room temperature. After incubation for 60 min in PBS/1% BSA, cells were washed in PBS/0.05% Triton X-100 and incubated with optimal dilutions of anti-Bax, anti-Tim23 primary antibodies and Hoechst for 60 min at room temperature (primary antibody IgG was diluted by PBS/0.05% Triton X-100 and 1% BSA). After three washes with PBS/0.05% Triton X-100, cells were incubated with the secondary antibodies for 60 min at room temperature: fluorescein isothiocyanate (FITC) conjugated goat anti-rabbit IgG (1:50) and Phycoerythrin (PE) conjugated donkey anti-mouse IgG (1:200). Cells were washed three times with PBS. Cells on cover slips were mounted with a slow-fade, anti-fade reagent onto glass slides and were observed under two-photon microscopy (Carl Zeiss, Thornwood, NY).

### Knockdown of PKR, RNase L and Bak

PC-3 cells were transfected with a heterogeneous mixture of 21–23 bp siRNA that induces effective silencing of PKR and RNase L by using a transpass R2 transfection reagent (New England Biolab, Ipswich, MA) according to the manufacturer's instructions. Cells were grown to 50% confluence. SiRNAs were incubated with the transfection reagent in a serum-free medium for 20 min at room temperature. Subsequently, the mixture was evenly dispersed onto the cells that have been washed once with serum-free medium. The final concentration of siRNAs was 12.5 nM. After incubation for 3 hours, the complete culture medium was added to the cells. The transfected cells were treated with IFN γ and poly I:C after 30 hours as described previously. The apoptotic effects of these treatments on the transfected PC-3 cells were analyzed. The expression of Bak in PC-3 cells was silenced by using a Bak siRNA (h) kit (sc-29786, Santa Cruz) in accordance to the manufacturer's instruction.

## SUPPLEMENTARY MATERIALS FIGURES


