# Correction: Effects of interferons and double-stranded RNA on human prostate cancer cell apoptosis

**DOI:** 10.18632/oncotarget.27257

**Published:** 2019-10-15

**Authors:** Haiyan Tan, Chun Zeng, Junbo Xie, Norah J. Alghamdi, Ya Song, Hongbing Zhang, Aimin Zhou, Di Jin

**Affiliations:** ^1^ Institute of Cancer Stem Cell, Dalian Medical University, Dalian, China; ^2^ Clinical Chemistry Program, Department of Chemistry, Cleveland State University, Cleveland, OH, USA; ^3^ Center for Gene Regulation in Health and Diseases, Cleveland State University, Cleveland, OH, USA; ^4^ College of Biotechnology and Food Science, Tianjin University of Commerce, Tianjin, China; ^5^ College of basic medical sciences, Dalian Medical University, Dalian, China

**This article has been corrected:** During processing of the data, the image sets of cells in Fig.1B were mistakenly combined. The correct Figure 1B is shown below. The authors declare that this correction does not change the results or conclusions of this paper.

Original article: 2015; 6:39184–39195. 39184-39195. https://doi.org/10.18632/oncotarget.5508

**Figure 1:**
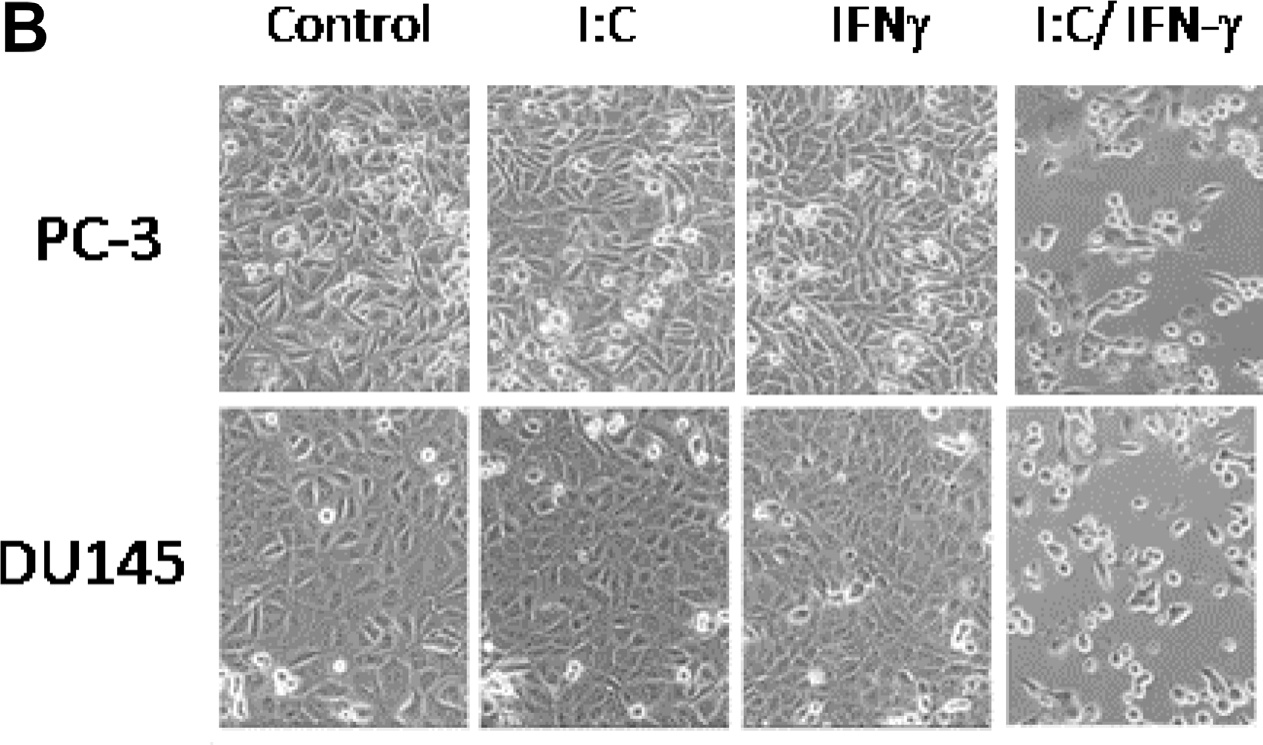
Effect of poly I:C and IFNs on PC-3 cell viability. PC-3 cells were treated with 1,000 unit/ml IFN α, β or γ overnight and transfected with and without 1 μg/ml poly I:C in the presence of lipofectamine. **A**. The viable cells were analyzed by trypan blue exclusion assays and the cell numbers were averaged from three independent experiments. Error bars represent ±SEM, and Student’s *t* test was used. ^*^ *p* < 0.05, ^**^ *p* < 0.01, ^***^ *p* < 0.001; and **B**. The pictures were taken under Olympus CKX31 at 100 × magnification.

