# Supplementary material for: Effects of interferons and double-stranded RNA on human prostate cancer cell apoptosis
Source: Oncotarget. 2015 Oct 1;6(36):39184–95. doi: 10.18632/oncotarget.5508 (PMC4770765; doi:10.18632/oncotarget.5508)
Supplement: Supplementary file 1 [file oncotarget-06-39184-s001.pdf]

SUPPLEMENTARY FIGURES

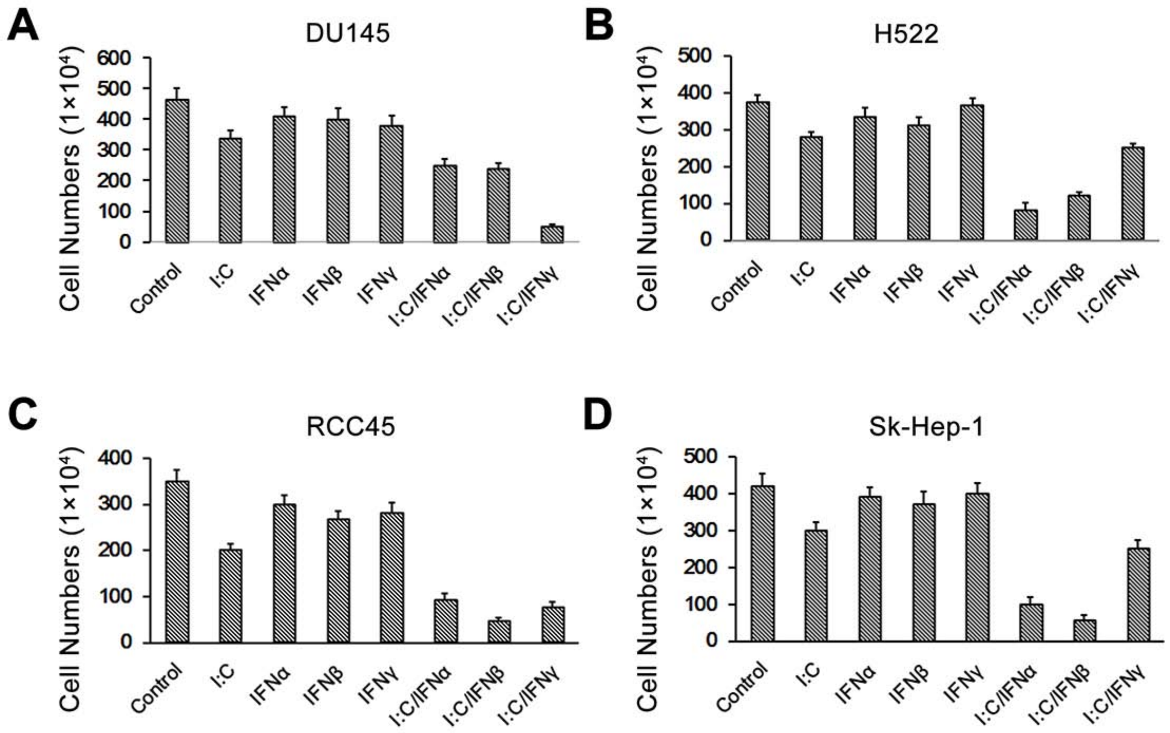

Supplementary Figure S1: Effect of poly I:C and IFNs on different cancer cell viability. Cancer cells were treated with 1,000 unit/ml human IFN α, β or γ overnight and transfected with and without 1 μg/ml poly I:C in the presence of lipofectamine. After incubation for 48 hours, the viable cells were analyzed by trypan blue exclusion assays and the cell numbers were averaged from two independent experiments. **A.** DU145 cells; **B.** H522 cells; **C.** RCC45 cells and **D.** Sk-Hep-1 cells. Error bars represent ± SEM.

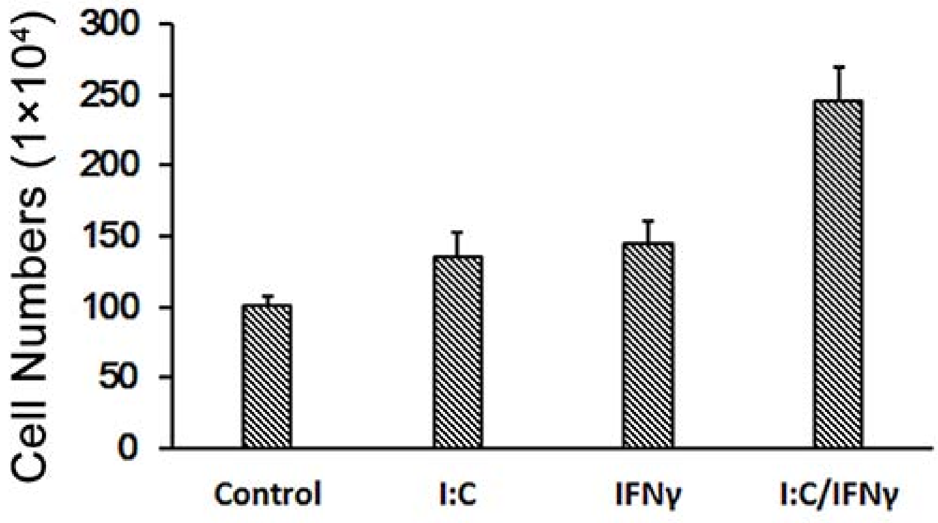

Supplementary Figure S2: Poly I:C and IFN γ-induced Caspase 3 activity in DU145 cells. DU145 cells were treated 1 μg/ml of poly I:C, 1,000 units of IFN γ alone, or a combination of both in the presence of lipofectamine for 24 hours. The activity of caspase 3 was measured using a Caspase-GloTM 3/7 assay kit (Promega, Madison, WI). Experiments were performed two times in triplicates. Error bars represent ± SEM.
